# Analysis of clinical features of myasthenia gravis complicated with hyperthyroidism

**DOI:** 10.12669/pjms.38.3.4656

**Published:** 2022

**Authors:** Yaxuan Wang, Guoyan Qi, Ying Yang

**Affiliations:** 1 Yaxuan Wang, Center of Treatment of Myasthenia Gravis Hebei Province, Shi Jiazhuang People’s Hospital, Shi Jiazhuang, 050000, Hebei, China; 2 Guoyan Qi, Center of Treatment of Myasthenia Gravis Hebei Province, Shi Jiazhuang People’s Hospital, Shi Jiazhuang, 050000, Hebei, China; 3 Ying Yang, Center of Treatment of Myasthenia Gravis Hebei Province, Shi Jiazhuang People’s Hospital, Shi Jiazhuang, 050000, Hebei, China

**Keywords:** Myasthenia gravis, Hyperthyroidism, Clinical features

## Abstract

**Objectives:**

To investigate the clinical features of patients with myasthenia gravis complicated with and without hyperthyroidism.

**Methods:**

A total of 2083 patients with myasthenia gravis (MG) admitted in Center of Treatment of Myasthenia Gravis Hebei Province between January 2013 and July 2020 were retrospectively analyzed and divided into two groups: Group-A and Group-B, with 108 MG patients complicated with hyperthyroidism in Group-A and 1975 MG patients without thyroid disease in Group-B. The age of onset, gender, Osserman classification, acetylcholine receptor antibody and thymus status of the two groups were analyzed in the two groups. Independent-sample t test was used for intra-group comparison, and χ2 test was utilized for comparison of enumeration data. P<0.05 indicates a statistically significant difference.

**Results:**

The age of onset in Group-A was significantly lower than that in Group-B (p=0.000), the number of female patients was significantly higher than that in Group-B (p=0.037), and the level of Achrabs titer was significantly lower than that in Group-B (p=0.000). The incidence of thymoma in Group-A was significantly lower than that in Group-B (p=0.012), while the incidence of thymic hyperplasia was significantly higher than that in Group-B (p=0.000).

**Conclusion:**

Patients with MG complicated with hyperthyroidism are mainly female, with a lower age of onset, a lower level of acetylcholine receptor antibody, a lower incidence of thymoma, and a higher incidence of thymic hyperplasia. The clinical features of such patients are remarkably different from those of MG without thyroid disease.

## INTRODUCTION

Myasthenia gravis (MG), as an autoimmune disease involving the postsynaptic membrane of the neuromuscular junction, is often accompanied by a variety of autoimmune diseases. MG is more associated with thyroid disease than with other autoimmune diseases.^1^ Thyroid disease autoimmune diseases in MG patients are mainly divided into three types: hyperthyroidism, hypothyroidism, and positive anti-thyroid antibodies^2^, among which hyperthyroidism is most frequently mentioned, which may have a close bearing on its high incidence and obvious clinical symptoms. MG and thyroid diseases both fall into autoimmune diseases, one of which bears a resemblance to the other in several aspects. Specifically, MG and thyroid diseases are both antibody-mediated diseases, which are organ specific, and can be manifested as abnormal ocular muscles. This may be related to the fact that both of them share a common genetic background.^3^ It has been shown in previous studies that the incidence of ocular MG is higher in cases of abnormal thyroid autoimmunity.^4^ However, the prevalence of thyroid disease varies from study to study, and it is not yet known whether the clinical features and course of disease in these patients are different from those in patients without hyperthyroidism. Therefore, the prevalence, age, gender, Osserman type, acetylcholine receptor antibody level and thymus condition of MG patients with hyperthyroidism in our center were evaluated and analyzed.

## METHODS

This is a retrospective analysis was conducted on 2,290 patients with MG who were admitted to and registered in the Center of Treatment of Myasthenia Gravis Hebei Province from January 2013 to July 2020. According to the inclusion criteria and exclusion criteria, a total of 2083 patients were included and divided into two groups: Group-A and Group-B, with 108 MG patients with hyperthyroidism (66 females, 42 male patients, aged 1-77 years old) in Group-A, and 1,975 MG patients without thyroid disease (1,003 females, 972 males, aged 4 months to 83 years old) in Group-B.

### Ethical approval

The study was approved by the Institutional Ethics Committee of Shi Jiazhuang People’s Hospital (No.:94; November 30, 2018), and written informed consent was obtained from all participants

### Diagnostic criteria:

- Typical skeletal muscle fatigue manifestations: eyelid drooping, diplopia, weakness of the limbs, difficulty in chewing and swallowing, difficulty in breathing, etc.
- Positive neostigmine test;
- Positive acetylcholine receptor antibody;
- Low-frequency amplitude of EMG repetitive nerve stimulation decreased by > 10%, while the amplitude of high frequency did not increase.^5^ Patients who met any 2 of the 4 criteria can be diagnosed as having MG.

### Diagnostic criteria for hyperthyroidism:

- Symptoms and signs of clinical hypermetabolism: subclinical hyperthyroidism may not be accompanied by symptoms or accompanied by mild symptoms;
- Thyroid gland signs: goiter and (or) thyroid nodules. A few cases may have no thyroid signs;
- Serum hormones: increased TT3, FT3, TT4 and FT4, and decreased TSH (generally < 0.1mIU/L). Generally, only increased TT3 and FT3 were observed in T3 hyperthyroidism, TSH level in subclinical hyperthyroidism was lower than the lower limit of normal, while TT3 and TT4 were normal.^6^ Patients who meet three criteria can be diagnosed as having hyperthyroidism.

### Clinical type of MG

MG type is based on the modified Osserman classification: type I: ocular muscle type; type IIA: mild systemic type; type 1I B: moderate systemic type; type Ill: severely aggressive type; type 1V: late-onset severe type; type V: muscular atrophy. Among them, type IIA-V were all systemic MG. Patients with MG were divided into the early-onset type (<50 years old) and the late-onset type (≥50 years old) according to the age of onset.

### Inclusion criteria

Patients who were definitely diagnosed with MG.

### Case exclusion criteria:

- Patients with hypothyroidism;
- Patients with normal thyroid function who suffer from positive thyroid peroxidase antibody and anti-thyroglobulin antibody;
- Patients with normal thyroid function who suffer from thyroiditis, thyroid nodules and thyroid cancer;
- Patients with previous thyroid-related surgery.

### Statistical methods

SPSS26.0 statistical software was used to analyze the data, independent sample t test was used for intra-group comparison, and χ2 test was utilized for comparison of enumeration data. P<0.05 indicates a statistically significant difference.

## RESULTS

The incidence of thyroid disease among patients admitted to our center was 13.8% (315/2290), of which the incidence of patients with hyperthyroidism was 4.7% (n=108).Sixty-six females and 42 males were assigned to Group-A, while 1003 females and 972 males were assigned to Group B (1.6:1 vs 1.03:1, p=0.037). It can be seen that the incidence of females in the two groups was relatively high, and that in Group-A was higher. Table-I. No significant difference was observed in the gender difference of patients in Group-A with any Osserman type, and the majority of patients were female. In Group-B, however, there were significantly more male patients with Osserman Type-I than female patients, and more female patients with type IIA-IV, showing a statistically significant difference in distribution. Table-II. Patients with MG were divided into the early-onset type (<50 years old) and the late-onset type (≥50 years old) according to the age of onset. In Group-A, females had a higher incidence, while in Group-B, MG of the late-onset type was more common in males, with no statistically significant difference. Table-I.

**Table-I:** Age and gender distribution of patients in different groups.

	A	B	p
n	108	1975	
F/M	66/42	1003/972	0.037
Age of onset of MG	28.75±18.9	37.77±23.23	0.000
F/M age	27.92±19.15/30.07±18.66 (p=0.7)	36.14±23.57/39.45±22.78 (p=0.002)	0.001/0.003
Type I age	25.17±19.269	27.45±26.17	0.532
Type II-IV age	32.22±18.048	44.58±18.12	0.000
Early-onset type/Late-onset type	86/22	1231/744	0.000
Early-onset type Female/Male	52/34	662/569	0.229 (NS)
Late-onset type Female/male	14/8	341/403	0.099 (NS)

NS: Not significant.

### Age differences

The age of onset of patients in Group-A was significantly lower than that of patients in Group-B (28.75±18.9 vs 37.77±23.23, p=0.000), while the age of onset of female and male were lower than those of Group-B (female: 27.92±19.15 vs 36.14±23.57, p=0.001, male 30.07±18.66 vs 39.45±22.78, p=0.003). Moreover, the onset age of females in both groups was lower than that of males, but statistically significant age difference was found between males and females in Group-B (female vs male: 36.14±23.57/39.45±22.78, p=0.002). There was no significant difference in the age of onset of muscle type, while the onset age of systemic type in Group-A was significantly lower than that in Group-B (32.22±18.048 vs 44.58±18.12, p=0.000).

### Clinical Type

According to the distribution of patients under Osserman type, the proportion of patients with Type-I in Group-A was significantly higher than that in Group-B, while the proportion of patients with type IIB-IV in Group-A was significantly less than that in Group-B, with statistically significant differences between the two groups (p=0.011). Table-II. However, there was no statistical difference in the distribution of ocular muscle type (I) and systemic type (IIA-IV) (p=0.054), and the number of patients with ocular muscle type was less than those with systemic type. Patients with early-onset type in Group-A were significantly more than those with late-onset type, with a statistical significance between the two groups (p=0.000). See Table-I.

**Table-II:** Comparison of Osserman type and gender distribution.

Osserman type	A (108)		B (1975)	

	n (%)	F/M	n (%)	F/M
I	53 (49.07%)	33/20	785 (39.47)	371/414
IIA	12 (11.11%)	6/6	131 (6.59%)	69/62
IIB	34 (31.48)	19/15	696 (34.99%)	365/331
III	8 (7.41%)	7/1	211 (10.61%)	114/97
IV	1 (0.92%)	1/0	152 (7.64%)	84/68
V	0 (0%)	0/0	0 (0%)	0/0
p=0.011		p=0.415		p=0.000

### Acetylcholine receptor antibodies

As shown in Table-III, there was no difference in the distribution of patients with positive AchRAbs between Group-A and Group-B (76.8% vs 81.9%, p=0.185). The AchRAbs titer level of Group-A was significantly lower than that of Group-B (5.24±4.75 vs 8.30±5.33, p=0.000).

**Table-III:** Comparison of acetylcholine receptor antibodies.

	A	B	p
AchRAbs (+) (%)	83 (76.8)	1618 (81.9)	0.185 (NS)
AchRAbs positive median	5.24±4.75	8.30±5.33	0.000

### Thymus

The incidence of thymoma in Group-A was significantly lower than that in Group-B (8.3% vs 17.8%, p=0.012). Based on postoperative pathological diagnosis, the incidence of thymic hyperplasia in Group-A was 24.1%, which was significantly higher than that in Group-B (7.3%), with a statistically significant difference between the two groups (p=0.000). Table-IV.

**Table-IV:** Thymus.

	A	B	p
Number of cases of thymoma (%)	9 (8.3)	351 (17.8)	0.012
Number of cases of thymic hyperplasia (%)	26 (24.1)	145 (7.3)	0.000

## DISCUSSION

It is particularly notorious that myasthenia gravis (MG) is an autoimmune disease and has been shown to be closely associated with thyroid disease in several previous studies. A possible association between MG and Graves’ disease was first reported in a paper^7^ in 1919. However, no sufficient evidence was provided in subsequent reports to indicate the relationship between the two diseases.^8^ It was found in a study published in 2011 that autoimmune thyroid disease is more frequent in female and MG patients with positive serum AChRAb.^1^ It was also shown in an article published by Petersson et al.^9^ in 2015 that MG patients suffer from an increased risk of another autoimmune disease. This risk is greater for young adults and females and may be regulated by the haplotype of HLA-B8-DR3. Graves’ disease has been confirmed to be associated with HLA-Dr antigen^10^, suggesting that MG and Graves’ disease are closely related in terms of genetic background. Moreover, patients with autoimmune diseases are susceptible to another or more autoimmune diseases, which may have a close bearing on their common genetic, environmental, and hormonal factors.^11^,^12^

In most previous studies, priority has been given to the correlation between thyroid disease and MG without exception. A systematic review of “Thyroid Disease in Patients with Myasthenia Gravis: Systematic Review and Meta-analysis”^13^ showed that approximately 10.1% of patients with MG were associated with thyroid autoimmune abnormalities: TgAb positive predominates, followed by GD and HT. The overall incidence of thyroid autoimmunity with thyroid dysfunction including hyperthyroidism and hypothyroidism in MG patients was 6.8%. A study in Taiwan showed that^14^ the incidence of autoimmune thyroid disease (ATD) was 8.2%, of which Graves’ disease accounted for the majority (5.7%), followed by antibody-positive thyroid disease and Hashimoto’s thyroiditis (1.4 % and 1.1%). However, thyroxine and thyroid antibody tests were not performed on all patients with MG in this study. As for the study on the correlation between hyperthyroidism and MG, only an epidemiological survey conducted by Peacey SR^12^ in 1993 showed that Graves’ disease is the most common thyroid disease associated with MG. It has also been proved that hyperthyroidism is more likely to be accompanied by MG than hypothyroidism^15^. In our study, emphasis was placed on exploring and comparing the clinical features of patients with hyperthyroidism complicated with MG (Group-A) and patients with MG without thyroid disease (Group-B). Each patient with MG was tested for thyroxine and thyroid antibodies at initial diagnosis. Of these cases, 4.7% had hyperthyroidism, and the incidence of thyroid disease was 13.8%. The age of onset of MG was earlier in Group-A (28.75 vs 37.77), and this phenomenon was found respectively in the comparison of the age of onset of female (27.92 vs 36.14) and male (30.07 vs 39.45) patients in Group-A and Group-B. As early as 1987, a study by Ohno M et al.^16^ in Japan has found that the average age of onset of MG symptoms in patients with Graves’ disease was less than that of patients with non-Graves’ disease (24.4 years and 35.5 years, respectively), which was the same as our findings.

In this study, statistical analysis were also made on the gender distribution of patients, and the results showed that there were more female patients, which was reflected in almost all Osserman subtypes. Only the patients with Type-I in Group-B had more male than female patients, indicating that patients with hyperthyroidism were more common in females. In terms of age of onset, the age of onset of females in Group-B was significantly earlier than that of males (36.14 vs 39.45). There was no significant age difference between males and females in Group-A. Studies have shown that there is a difference between the peak incidence of male and female. Females reach a peak around 30 years old, while males have two peak incidences at 30 and 60 years old.^17^ Therefore, the incidence of early-onset type and late-onset type MG was compared between male and female, and it was found that the number of early-onset type patients was significantly more than that of late-onset type patients in Group-A, and the number of both early-onset type and late-onset type female was more than that of male, which may be related to the tendency of young female to develop hyperthyroidism. In Group-B, the number of male patients with late-onset type was more than that of female patients, which may be related to two morbidity peaks in male patients. Myasthenia gravis was classified as Oseerman I, IIa, IIb, III, IV, and V based on the patient’s clinical manifestations.

A number of studies have shown that the clinical features and treatment methods of myasthenia gravis vary between the ocular muscle type and the systemic type. In our study, the distribution of patients with ocular muscle type and systemic type was compared between the two groups, and there was no statistically significant difference. No significant difference was observed in the age of onset of ophthalmic muscle type between the two groups. The age of onset of systemic type in Group-A was significantly lower than that in Group-B, and the incidence of III and IV in Group-A was significantly lower than that in Group-B (7.4%vs18.3%), suggesting that patients in Group-A had mild clinical symptoms and were less likely to develop myasthenic crisis. In addition, in this study, no significant difference was found in the positive rate of ACHRAB between the two groups, and the titer level of Group-A was lower than that of Group-B, which may be related to young patients and mild and stable clinical symptoms.^18^ It was found by Martignago et al.^19^ in 2009 that ACR-Ab seropositive Mg is associated with thymoadenoma or thymic hyperplasia and thyroid diseases. Thus, autoimmunity plays a crucial role in the association between MG and thyroid diseases. In this study, statistics were performed on the patient’s thymus. Taking pathology after surgery as the diagnostic criteria, the probability of thymic hyperplasia in patients with hyperthyroidism is significantly higher than that in patients without thyroid disease. On the contrary, the incidence of thymoma in patients with MG complicated with hyperthyroidism is 8.3%, which was significantly lower than the incidence of thymoma in patients with myasthenia gravis without thyroid disease (17.8%), with statistically significant results. The correlation between thymoma and MG has been confirmed, and previous studies have also confirmed a close relationship between thyroid disease and MG.^11^,^12^,^15^,^20^ Previous researchers have conducted studies on the thymic hyperplasia rate in patients with myasthenia gravis with thyroid disease, and the results are different^4^,^21^,^22^, which may be related to the number of cases, diagnosis basis and other factors.Previous studies have been conducted on the rate of thymus hyperplasia in patients with MG complicated with thyroid diseases, and the results are different,^4^,^21^,^22^ which may be related to various factors such as the number of statistical cases and the diagnostic basis.

However, no significant association between thymoma and thyroid gland has been reported in any study. In view of this, the difference in the analysis results in our study may be attributed to a variety of factors, such as myasthenia gravis patients with hyperthyroidism are younger, with milder symptoms and earlier clinical classifications, while thymoma occurs mostly in 30-50-year-old patients with myasthenia gravis have severe symptoms and late classification.

### Limitations of the study

Nevertheless, deficiencies can be seen in this study: the results of this study are limited due to the fact that it was a retrospective rather than a case-control study. In view of this, proactive countermeasures will be taken in the future to carry out more comprehensive studies on such patients, so that more scientific data can be made available to our clinicians.

## CONCLUSION

Patients with MG complicated with hyperthyroidism and those without thyroid disease have great differences in clinical features, and the possibility of ethnic differences could not be ruled out. Moreover, thyroid function testing combined with thyroid ultrasound examination is indispensable for every patient with MG, which is conducive for early detection of MG patients with asymptomatic thyroid disease so as to further carry out more targeted clinical treatment.

### Authors’ Contributions:

**YW:** Designed this study and prepared this manuscript, and are responsible and accountable for the accuracy or integrity of the work.

**GQ:** Collected and analyzed clinical data.

**YY:** Significantly revised this manuscript.
